# Kudzu Celery Decoction Exerts Protection against Sepsis-Induced Myocardial Injury

**DOI:** 10.1155/2022/2886932

**Published:** 2022-05-04

**Authors:** Lin Zhao, Huadong Zhao, Meng Sun, Mengfan Chen, Xue Wu, Chao Deng, Wenwen Yang, Ye Tian, Qibing Wang, Zhenxing Liang, Xuezeng Xu, Yang Yang

**Affiliations:** ^1^Xi'an Key Laboratory of Cardiovascular and Cerebrovascular Diseases, Xi'an No. 3 Hospital, The Affiliated Hospital of Northwest University, School of Life Sciences and Medicine, Northwest University, 10 Fengcheng Three Road, Xi'an, China; ^2^Department of Cardiovascular Surgery, Xijing Hospital, The Fourth Military Medical University, Xi'an, China; ^3^Key Laboratory of Resource Biology and Biotechnology in Western China, Ministry of Education, Faculty of Life Sciences, Northwest University, Xi'an, China; ^4^Department of General Surgery, Tangdu Hospital, The Fourth Military Medical University, Xi'an, China; ^5^Department of Cardiology, The First Hospital of Shanxi Medical University, Taiyuan, China; ^6^Department of Cardiovascular Surgery, The First Affiliated Hospital of Xi'an Jiaotong University, Xi'an, Shaanxi, China; ^7^Department of Cardiothoracic Surgery, The First Affiliated Hospital of Zhengzhou University, Zhengzhou, China

## Abstract

Myocardial dysfunction is well-recognized manifestations of organ dysfunction in sepsis, which is the leading cause of death in critically ill patients. The underlying mechanisms associated with sepsis-induced myocardial injury (SIMI) include cardiac contractility, inflammatory response, oxidative stress, and apoptosis. Kudzu celery decoction (KCD) is composed of a variety of traditional Chinese medicine (TCM) such as kudzu and celery. The previous study found that the main ingredients in kudzu and celery have also been proved to have anti-inflammatory, antioxidative, and other biological activities. In this study, the therapeutic effects of KCD were evaluated in the cecal ligation and puncture (CLP) model of BALB/c mice. The effects of KCD on cardiac function, myocardium damage, inflammation, and fibrosis in CLP-injured mice were analyzed with echocardiography, histological staining, and quantitative real-time PCR. The results showed that KCD treatment improved the anal temperature, sepsis score, blood routine parameters, and blood biochemical parameters in CLP-injured mice. Then, we observed that KCD could remarkably alleviate cardiac dysfunction, myocardial fibrosis, oxidative stress, and inflammation in CLP-injured mice. In this study, we confirmed that KCD has a significant protective effect on SIMI, which may favor KCD a potential cardioprotective drug candidate to alleviate SIMI and further amplify the application of TCM prescription in clinic.

## 1. Introduction

The Third International Consensus Definition for Sepsis and Septic Shock (Sepsis-3.0) defined that sepsis is a life threaten organ dysfunction caused by the host's dysfunctional response to infection in 2016 [1]. Clinical studies have confirmed that sepsis causes multiple organ dysfunction syndrome with extremely high mortality rate and seriously threatens human health [2]. Myocardial dysfunction is a recognized manifestation of sepsis and septic shock, which leads to insufficient perfusion of blood flow to various organs throughout the body and significantly increases the occurrence of sudden cardiovascular events [3]. Studies have confirmed that the underlying mechanisms associated with sepsis-induced myocardial injury (SIMI) include myocardial systolic dysfunction, inflammatory response, oxidative stress, myocardial fibrosis, and apoptosis [4, 5]. However, the specific pathogenic mechanism of SIMI has not been fully elucidated. Moreover, numerous studies have attempted to identify agents that are protective against SIMI, but very few candidates have achieved profoundly beneficial effects.

The main ingredients of kudzu celery decoction (KCD) are kudzu, celery, hawthorn, cassia seed, Chinese yam, Fructus lycii, stigma maydis, and lactose. Puerarin is an isoflavone extracted from kudzu roots and has been reported to possess antioxidative, anti-inflammatory, antiapoptotic, and antidiabetic properties [6]. Previous studies have shown that kudzu extract and its bioactive isoflavones could be effective for multiple cardiovascular diseases such as hypertension, atherosclerosis, and myocardial infarction in clinical therapy [7]. Moreover, celery has abundant flavonoid luteolin, which exerts prominent antioxidative and anti-inflammatory activities [8]. The KCD was originally developed by Shaanxi Health Chi Biological Pharmaceutical Co., Ltd., and passed the safety standard requirements under the instructions of the inventor, and obtained the National Science and Technology Commission utility patent number 202110493275.3. Moreover, the product has formed a solid beverage in China and passed the national certification (product standard number: Q/SXJCNNN0001S and production license: SC10661990500017). Meanwhile, the company has demonstrated that KCD not only lower blood pressure but also reduce triglycerides and cholesterol, restore vascular elasticity, and improve arteriosclerosis. Moreover, they also found KCD can effectively reduce the blood glucose of diabetic patients (China Patent Application No: CN201510070734.1). However, whether KCD can exert beneficial effects on SIMI has not been elucidated yet. Therefore, the main purpose of this study is to investigate whether KCD has protective effects on SIMI. It also provides a theoretical basis for KCD as a clinical cardioprotective drug in the future.

## 2. Materials and Methods

### 2.1. Animals

**Figure 1:**
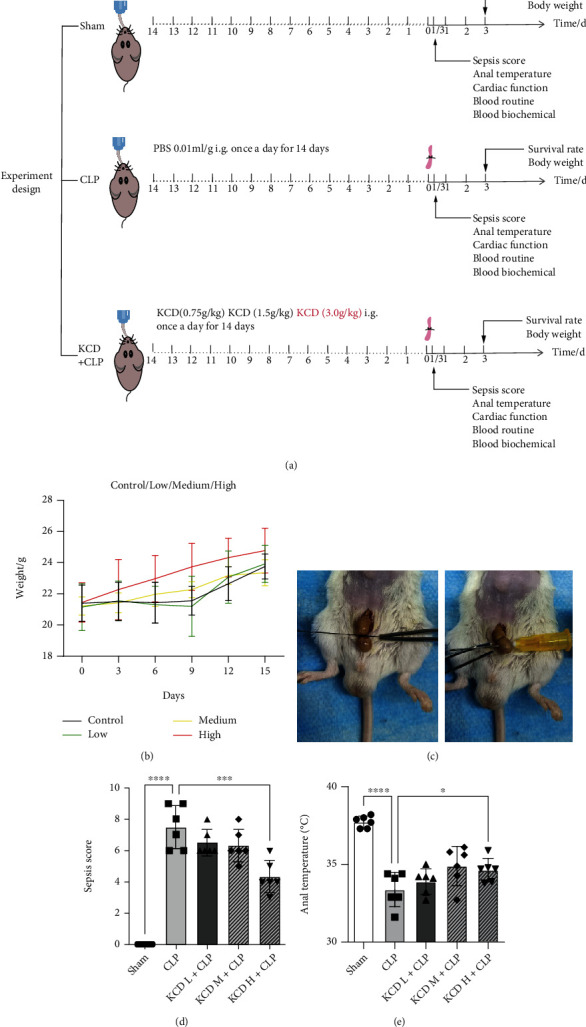
The effects of KCD on body weight, sepsis sore, and anal temperature in CLP-injured mice. (a) A schematic diagram of the experimental group and protocol. (b) Body weight analysis of normal mice within 15 days. (c) The picture of the CLP modeling process. (d) Sepsis score. (e) Anal temperature. ^∗^*P* < 0.05, ^∗∗∗^*P* < 0.001, ^∗∗∗∗^*P* < 0.0001 vs. CLP group; ns: nonsignificant; *n* = 6.

Male BALB/c mice (22-25 g) aged 8-10 weeks were obtained from the animal center of PLA Air Force Military Medical University (Xi'an, Shaanxi, China). All animal experiment protocols were performed in accordance with the guidelines of Animal Care and Use Committees at Northwest University (Xi'an, Shaanxi, China) and were in compliance with the Guidelines for the Care and Use of Laboratory Animals (NIH Publication No. 85–23, revised 1996). All mice had free access to food and water and were bred at 26°C in a 12 h light/12 h dark cycle. The main ingredients of KCD are kudzu (8-12 parts), celery (6-8 parts), hawthorn (15-25 parts), cassia seed (15-25 parts), Chinese yam (6-8 parts), Fructus lycii (3-6 parts), stigma maydis (5-8 parts), and lactose. To determine the optimum protective effects of KCD (Health Chi Biological Pharmaceutical Co., Ltd., Xi'an, Shaanxi, China) on CLP-injured mice, KCD (0.75, 1.5, and 3 g/kg, i.g.), dissolved in phosphate buffer salt solution (PBS), was given once a day for 15 days (Figure 1(a)).

### 2.2. Cecal Ligation and Puncture (CLP) Model

All mice were fasted for 8 h before the operation, but water was allowed ad libitum. Mice were anesthetized with 2% inhaled isoflurane. The abdominal hair of mice was shaved off, and the abdominal skin of mice was sterilized with 75% alcohol. A 1-2 cm midline laparotomy was performed to expose the cecum with adjoining intestine. The cecum was tightly ligated at 1/3 site from its end using 4-0 nylon suture, and double punctures of the cecal wall were performed with a 25 G needle. A small droplet of feces was squeezed through the puncture site to ensure patency, and it was returned to the peritoneal cavity, then the incision of peritoneum, fasciae, abdominal musculature, and skin was sutured with a sterile 6-0 silk. Except for cecum ligation and perforation, the other operations in the sham operation group were the same as those in the operation group. All operated mice were resuscitated by injecting prewarmed normal saline (1 mL/100 g, 37°C) subcutaneously. Sepsis score was conducted by two investigators after the induction of sepsis for 8 h by murine sepsis score (MSS). The MSS system involves observing seven components: appearance, level of consciousness, activity, response to stimulus, eyes, respiratory rate, and respiratory quality. Each of these variables are given a score between 0 and 4 and are referred with Shrum et al. [9]. The established MSS score is the total of these seven components. Subsequently, the anal temperature was determined at post-CLP 8 h with the animal thermometer (Calvin Biotechnology Co., Ltd., Nanjing, Jiangsu, China).

### 2.3. Detection of Blood Routine Parameters and Blood Biochemical Parameters

At 8 h post-CLP, at least 10 *μ*L blood was collected from the left eyeball into a heparin-coated tube. The levels of white blood cells (WBC), red blood cells (RBC), platelets (PLT), and lymphocytes (LYM) were detected by an automatic blood analyzer (Genrui Technology Co., Ltd., KT6200VET, Shenzhen, Guangdong, China). After that, 150 *μ*L serum was isolated from the rest of the whole blood by being centrifuged at 3000 rpm for 10 min. The levels of CK (creatine kinase), aspartate aminotransferase (AST), albumin (ALB), and blood urea nitrogen (BUN) were detected by an automatic blood biochemical analyzer (XinRui Technology Co., Ltd., XR210, Zhongshan, Guangdong, China).

### 2.4. Echocardiography Evaluation

Transthoracic echocardiography was performed using an animal-specific instrument (VisualSonics Vevo3100, VisualSonics, Toronto, ON, Canada) at 8 h post-CLP in mice. Mice were anaesthetized with 2% isoflurane in an induction chamber for 1-2 min. Next, mice were, respectively, laid supine on a warm platform and kept anesthetized by 2% isoflurane until they lost body-righting reflex. Then, a series of M-mode images at the level of papillary muscles were obtained. Stroke volume (SV), cardiac output (CO), left ventricular posterior wall thickness of systole period (LVPWs), left ventricular posterior wall thickness of diastole period (LVPWd), left ventricular diastolic volume (LVEDV), and left ventricular systolic volume (LVESV) were measured using Vevo LAB 3.0.0. All measurements were based on three consecutive cardiac cycles.

### 2.5. Histological Stating

To analyze the histopathological changes in the myocardium, the heart was excised, and one part of the myocardium was fixed overnight in 4% paraformaldehyde, embedded in paraffin, and dehydrated in an ascending series of ethanol (70, 80, 90, and 100%). The tissue samples were embedded in paraffin wax and cut into 4-5 *μ*m thick sections. The sections were mounted on standard glass slides and stained with hematoxylin and eosin (H&E) for 2 min before histological examination. Images of the stained sections were obtained using a light microscope (EVOSM5000, Thermo Fisher Scientific, Carlsbad, CA, USA). The degree of myocardial fibrosis was examined by Masson staining (Solarbio, Co., Ltd., Beijing, China). For immunostaining, paraffin-embedded slices were stained with the respective primary antibodies against Ly6G (1 : 200, Servicebio, Wuhan, China), F4/80 (1 : 200, Servicebio, Wuhan, China), NOX2 (1 : 200, Servicebio, Co., Ltd., Wuhan, Hubei, China), and 8-OHdg (1 : 200, Santa Cruz Biotechnology, Dallas, TX, USA), then incubated with a secondary biotinylated anti-rabbit IgG, stained with 3,3′-diaminobenzidine (DAB), and imaged using a microscope (Invitrogen EVOS M5000, Thermo Fisher Scientific, Waltham, MA, USA). Finally, IHC were quantified using Image-Pro plus 6.0 software.

### 2.6. Quantitative Real-Time PCR (qRT-PCR)

Total RNA was extracted from myocardium using the TRIzolTM total RNA Extraction Kit (Takara Bio Inc. Kusatsu, Shiga, Japan), and reverse transcription was performed using the Prime Script RT Master Mix (Takara Bio Inc. Kusatsu, Shiga, Japan). Then, NLRP3, IL-1*β*, IL-6, TNF-*α*, and CXCL2 mRNA levels were detected using quantitative real-time reverse transcriptase PCR analysis with SYBR Premix Ex Taq in Table 1 (Hunan Accurate Biotechnology Co., Ltd., Changsha, Hunan, China). The reaction conditions were as follows: (1) 95°C for 10 min, (2) 40 cycles of 95°C for 5 s and 60°C for 30 s, (3) 94°C for 30 s, 60°C for 90 s, and 94°C for 10 s. The expression levels of the examined transcripts were compared to that of *β*-actin and normalized to the mean value of the controls.

### 2.7. Statistical Analysis

Data were analyzed by using GraphPad Prism 9.0.0 (GraphPad Software Inc., San Diego, CA, USA). All values are presented as the mean ± standard deviation (SD). For the difference analysis of experimental data, *t* test was used for any two groups, and one-way ANOVA was used for more than two groups. *P* < 0.05 indicated that the date had significant differences.

## 3. Results

### 3.1. The Effects of KCD on Body Weight of Normal Mice, Sepsis Score, and Anal Temperature in CLP-Injured Mice

First, the body weight of the mice was observed every day. KCD, especially at high doses, slightly increased the body weight of normal mice, but there was not a significant difference (compared with the control group, Figure 1(b)). CLP is one of the most widely used model for experimental sepsis. The establishment of CLP model mainly consists of two steps: cecal ligation and puncture (Figure 1(c)). Moreover, the sepsis score and anal temperature were performed 8 h post CLP. As shown in Figure 1(e), KCD treatment significantly increased the anal temperature in CLP-injured mice (compared with the CLP group, *P* < 0.05). In this study, a murine sepsis score system was introduced to monitor the mice based on their appearance, level of consciousness, activity, response to stimuli, eyes, respiration rate and quality (from 0 to 4 points for each criteria). The results showed that CLP significantly increased sepsis score, while KCD treatment reversed these results (compared with the CLP group, Figure 1(d), *P* < 0.05).

### 3.2. The Effects of KCD on Blood Routine Parameters and Blood Biochemical Parameters in CLP-Injured Mice

**Figure 2:**
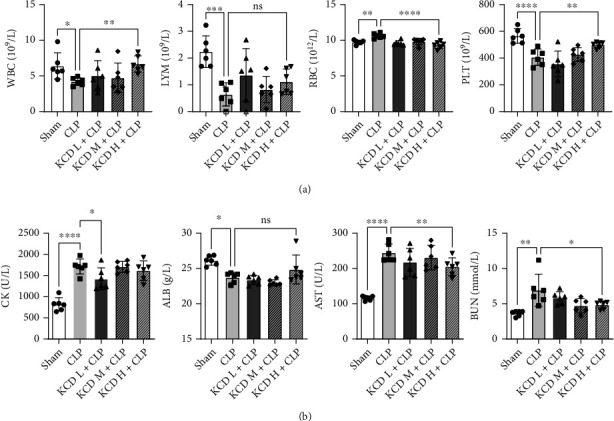
The effects of KCD on blood routine parameters and blood biochemical parameters in CLP-injured mice. (a) Blood routine parameters. (b) Blood biochemical parameters. ^∗^*P* < 0.05, ^∗∗^*P* < 0.01, ^∗∗∗^*P* < 0.001, ^∗∗∗∗^*P* < 0.0001 vs. CLP group; ns: nonsignificant; *n* = 6.

Then, blood routine parameters and blood biochemical parameters were detected. Compared with the CLP group, KCD remarkably increased the levels of WBC and PLT and decreased the level of RBC (Figure 2(a), *P* < 0.05), whereas KCD had no significant effect on LYM level in the blood. In addition, CLP injury markedly rose the levels of blood biochemical parameters (CK, BUN and AST) and decrease the level of ALB. However, KCD treatment could reverse these alters except for ALB (Figure 2(b), *P* < 0.05). The protective effect was obvious with high dose of KCD, so this concentration of KCD was adopted for further studies.

### 3.3. The Effects of KCD on Cardiac Function and Myocardial Fibrosis in CLP-Injured Mice

**Figure 3:**
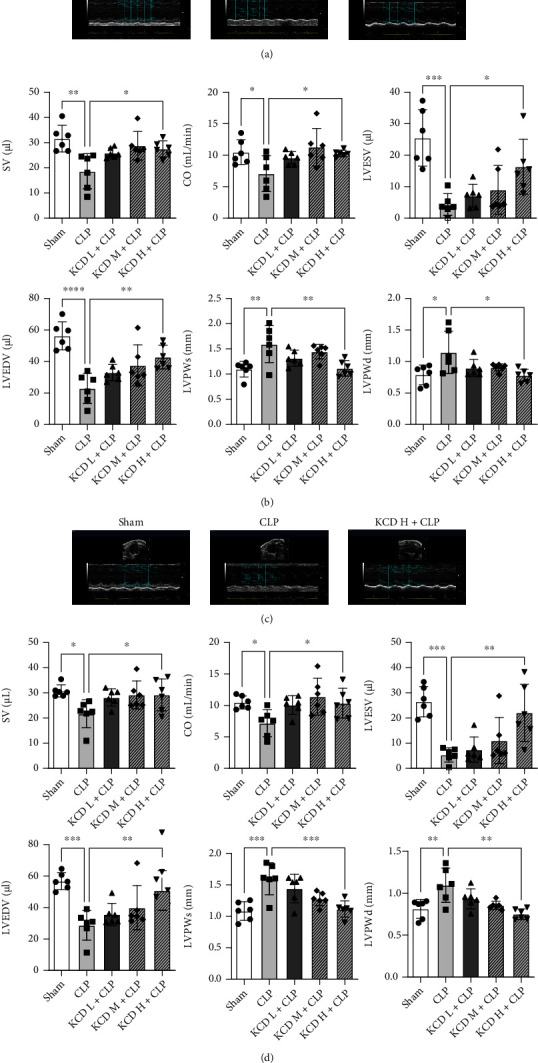
The effects of KCD on cardiac function in CLP-injured mice. (a) Representative echocardiography images of the long axis, *n* = 6. (b) Statistical graph of SV, CO, LVESV, LVEDV, LVPWs, and LVPWd. (c) Representative echocardiography images of the short axis, *n* = 6. (d) Statistical graph of SV, CO, LVESV, LVEDV, LVPWs, and LVPWd. ^∗^*P* < 0.05, ^∗∗^*P* < 0.01, ^∗∗∗^*P* < 0.001, ^∗∗∗∗^*P* < 0.0001 vs. CLP group; ns: nonsignificant.

**Figure 4:**
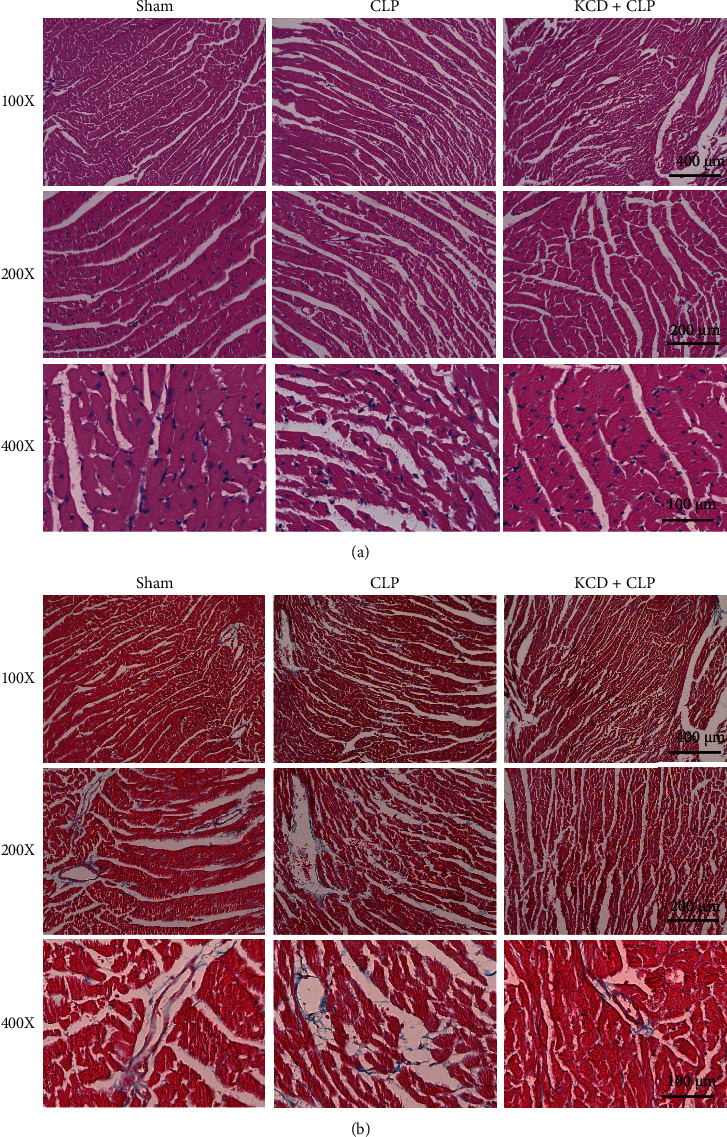
The effects of KCD on myocardial structure and myocardial fibrosis in CLP-injured mice. (a) H&E staining, *n* = 3. (b) Masson staining, *n* = 3.

The indicators of cardiac function were detected by echocardiography. As shown in Figures 3(a) and 3(b), CLP resulted in severe myocardial injury in long axis view, as evidenced by decreased SV, CO, LVESV, and LVEDV and increased LVPWs and LVPWd (compared with the sham group, *P* < 0.05). KCD treatment showed obvious cardioprotective effect by restoring these parameters of cardiac function (compared with the CLP group, *P* < 0.05). Results from the short axis view took on the same trend (Figures 3(c) and 3(d)). Besides, H&E staining and Masson staining have shown obvious structural abnormalities and myocardial fibrosis in CLP-injured myocardium, while KCD treatment remarkably reversed these pathological changes induced by CLP (Figures 4(a) and 4(b)).

### 3.4. The effects of KCD on structure and fibrosis in CLP-injured mice.

**Figure 5:**
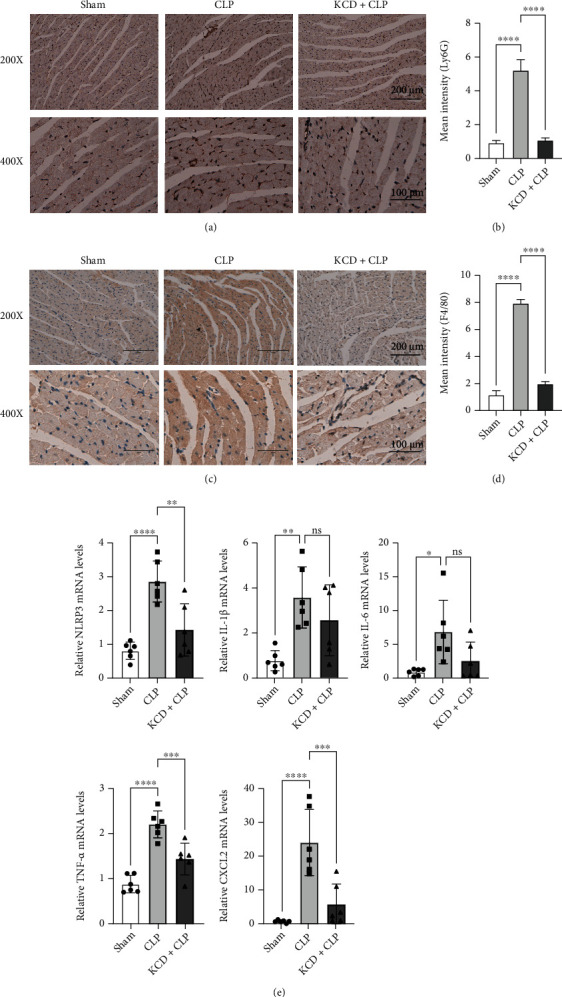
The effects of KCD on inflammatory response in CLP-injured mice. (a) The representative images of Ly6G IHC staining, *n* = 3. (b) Statistical graph of Ly6G IHC staining. (c) The representative images of F4/80 IHC staining, *n* = 3. (d) Statistical graph of F4/80 IHC staining. (e) qRT-PCR analysis of NLRP3, IL-1*β*, IL-6, TNF-*α*, and CXCL2, mRNA by normalizing to *β*-actin, *n* = 6. ^∗^*P* < 0.05, ^∗∗^*P* < 0.01, ^∗∗∗^*P* < 0.001, and ^∗∗∗∗^*P* < 0.0001 vs. CLP group; ns: nonsignificant.

**Figure 6:**
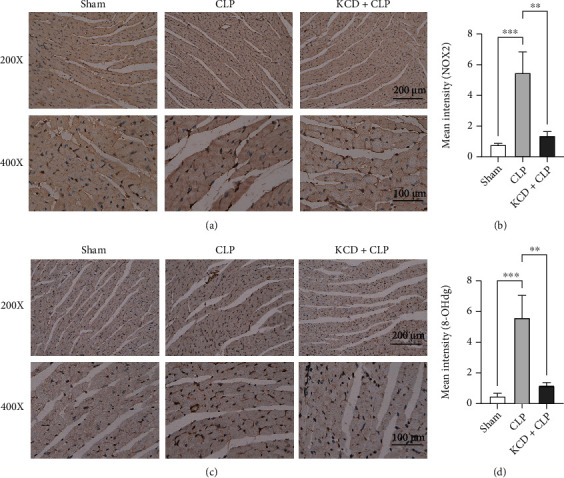
The effects of KCD on oxidative stress in CLP-injured mice. (a) The representative images of NOX2 IHC staining, *n* = 3. (b) Statistical graph of NOX2 IHC staining. (c) The representative images of 8-OHdg IHC staining, *n* = 3. (d) Statistical graph of 8-OHdg IHC staining. ^∗∗^*P* < 0.01, ^∗∗∗^*P* < 0.001 vs. CLP group; ns: nonsignificant.

As shown in Figure 5(e), the qRT-PCR assay demonstrated that CLP injury significantly increased NLRP3, IL-1*β*, IL-6, TNF-*α*, and CXCL2 mRNA expressions. However, KCD treatment could reverse these effects except for IL-1*β* and IL-6 (Figure 5(e), *P* < 0.05). Moreover, the IHC staining of Ly6G and F4/80 was also performed to visualize their expressions in the myocardium. As expected, KCD significantly attenuated the inflammatory response, as indicated by decreased expressions of Ly6G and F4/80 (compared with the CLP group, *P* < 0.05, Figures 5(a)–5(d)). In addition, this study also investigated the effect of KCD on the expression of oxidative stress markers (NOX2 and 8-OHdg) in the myocardium. The results showed that KCD significantly attenuated the oxidative stress, as indicated by decreased expressions of NOX2 and 8-OHdg (compared with the CLP group, *P* < 0.05, Figures 6(a)–(d)).

## 4. Discussion

**Figure 7:**
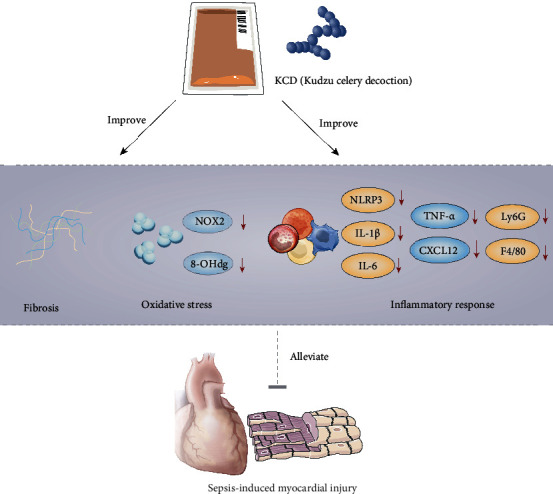
Proposed scheme depicting the mechanisms of KCD protecting against sepsis-induced myocardial injury.

Sepsis is a comprehensive disease with high mortality and is characterized by systemic inflammatory response syndrome. The clinical manifestations of sepsis are various complications. SIMI is closely related to sepsis mortality rate [10]. Previous studies have shown several progress with great significance in SIMI, including extravagant inflammatory response, apoptosis, and fibrosis [10]. It is reported that the main ingredients of KCD (kudzu and celery) have various biological activities such as anti-inflammatory and antioxidative, which are responsible for its extensive therapeutic effects [11, 12]. In this study, we found that KCD exerted significantly protective effects against SIMI by alleviating inflammation, oxidative stress, and myocardial fibrosis (Figure 7).

Inflammatory response plays an important role in myocardial injury, and the production of proinflammatory cytokines is a key step in SIMI [13]. Clinical and experimental studies have shown that the massive release of proinflammatory cytokines (IL-6 and TNF-*α*) can trigger sepsis-induced cardiac dysfunction [14], and inhibition of the expressions of IL-6 and TNF-*α* can significantly attenuate SIMI [15]. Zeng et al. found that CLP-injured can increase the levels of IL-1*β*, IL-6, and TNF-*α* in the heart of septic mice [16]. In this study, KCD significantly reduced NLRP3, IL-1*β*, IL-6, TNF-*α*, and CXCL2 mRNA expressions. Neutrophils are the firstly recruited cells that release chemotactic factors and elicit the invasion of monocytes into the injured myocardium; Ly6G are often used as marker of neutrophil in these conditions. Additionally, F4/80, specific macrophage marker, are known to play an important role in apoptosis, clearing damaged tissue and wound healing, particularly in the heart. Therefore, to ascertain the effect of KCD on neutrophil infiltration into myocardium, we examined the Ly6G and F4/80 expression by IHC staining in myocardium of CLP-injured mice. We also found that KCD significantly attenuated the inflammatory response, as indicated by decreased expressions of Ly6G and F4/80.

Myocardial fibrosis, an important hallmark of maladaptive hypertrophy, plays a critical role in SIMI. Myocardial fibrosis is characterized by the excessive accumulation of collagens and other extracellular matrix (ECM), resulting in myocardial stiffness, cardiac remodeling, and eventual heart failure [17]. Zhang et al. found that CLP injury significantly induced cardiac perivascular and interstitial fibrosis in septic mice [18]. Moreover, Tomita et al. demonstrated that ONO-4817 (MMP inhibitor) can obviously inhibit the rapid progression of myocardial fibrosis in septic mice [19]. In this study, KCD treatment could alleviate myocardial fibrosis by decreasing the deposition of collagen fiber in CLP-injured mice.

## 5. Conclusion

In conclusion, this study demonstrates for the first time the beneficial effects of KCD on SIMI, which is manifested in amelioration of inflammatory response, oxidative stress, and myocardial fibrosis (Figure 7). Therefore, these finding may favor KCD a potential cardioprotective drug candidate to alleviate SIMI in clinical.

## Figures and Tables

**Table 1:** Sequence of primers used in PCR amplification.

Gene		Sequence
NLRP3	Forward	GGAGTTCTTCGCTGCTATGTA
	Reverse	GGACCTTCACGTCTCGGTTC
IL-1*β*	Forward	GTGTCTTTCCCGTGGACCTT
	Reverse	CATCTCGGAGCCTGTAGTGC
IL-6	Forward	TTGGGACTGATGCTGGTGAC
	Reverse	GGTATAGACAGGTCTGTTGGGAGT
TNF-*α*	Forward	ACTGAACTTCGGGGTGATCG
	Reverse	TGGTGGTTTGCTACGACGTG
CXCL2	Forward	CCACCAACCACCAGGCTACA
	Reverse	CTGTAGCCTGGTGGTTGGT

## Data Availability

The original contributions presented in the study are included in the article; further inquiries can be directed to the corresponding authors.

## Abbreviations

SIMI: Sepsis-induced myocardial injury
KCD: Kudzu celery decoction
CLP: Cecal ligation and puncture
WBC: White blood cells
RBC: Red blood cells
PLT: Platelets
LYM: Lymphocytes
CK: Creatine kinase
AST: Aspartate aminotransferase
ALB: Albumin
BUN: Blood urea nitrogen
SV: Stroke volume
CO: Cardiac output
LVPWs: Left ventricular posterior wall thickness of systole period
LVPWd: Left ventricular posterior wall thickness of diastole period
LVEDV: Left ventricular diastolic volume
LVESV: Left ventricular systolic volume
HR: Heart rate
H&E: Hematoxylin-eosin
ECM: Extracellular matrix.
